# Minimum Determinants of Transmissible Gastroenteritis Virus Enteric Tropism Are Located in the N-Terminus of Spike Protein

**DOI:** 10.3390/pathogens9010002

**Published:** 2019-12-18

**Authors:** Carlos M. Sanchez, Alejandro Pascual-Iglesias, Isabel Sola, Sonia Zuñiga, Luis Enjuanes

**Affiliations:** Department of Molecular and Cell Biology, National Center of Biotechnology (CNB-CSIC), Campus Universidad Autónoma de Madrid, Darwin 3, 28049 Madrid, Spain; csanchez@cnb.csic.es (C.M.S.); alejandro.pascual@cnb.csic.es (A.P.-I.); isola@cnb.csic.es (I.S.)

**Keywords:** transmissible gastroenteritis virus, tropism, recombinant virus, enteric tropism

## Abstract

Transmissible gastroenteritis virus (TGEV) is an enteric coronavirus causing high morbidity and mortality in porcine herds worldwide, that possesses both enteric and respiratory tropism. The ability to replicate in the enteric tract directly correlates with virulence, as TGEVs with an exclusive respiratory tropism are attenuated. The tissue tropism is determined by spike (S) protein, although the molecular bases for enteric tropism remain to be fully characterized. Both pAPN and sialic acid binding domains (aa 506–655 and 145–155, respectively) are necessary but not sufficient for enteric tract infection. Using a TGEV infectious cDNA and enteric (TGEV-SC11) or respiratory (TGEV-SPTV) isolates, encoding a full-length S protein, a set of chimeric recombinant viruses, with a sequential modification in S protein amino terminus, was engineered. In vivo tropism, either enteric, respiratory or both, was studied by inoculating three-day-old piglets and analyzing viral titers in lung and gut. The data indicated that U655>G change in S gene (S219A in S protein) was required to confer enteric tropism to a respiratory virus that already contains the pAPN and sialic acid binding domains in its S protein. Moreover, an engineered virus containing U655>G and a 6 nt insertion at position 1124 (Y374-T375insND in S protein) was genetically stable after passage in cell cultures, and increased virus titers in gut by 1000-fold. We postulated that the effect of these residues in enteric tropism may be mediated by the modification of both glycosaminoglycan binding and S protein structure.

## 1. Introduction

Porcine enteric coronaviruses (CoVs) are one of the main threats for porcine industry worldwide, as acute infectious diarrhea is a major cause of high morbidity and mortality in piglets [1]. Transmissible gastroenteritis virus (TGEV) is one of the five enteric porcine CoVs described so far [2]. TGEV is classified in subgenus *Tegacovirus* of genus *Alphacoronavirus*, included in the family *Coronaviridae* into the order *Nidovirales* [3,4]. CoVs contain the largest known genome among RNA viruses, consisting in a single-stranded, positive-sense, 5′-capped and polyadenylated RNA molecule of around 28 kb in length [5]. TGEV genomic RNA (gRNA) encodes replicase polyproteins (pp1a and pp1ab), and a set of structural and accessory genes in the order 5′-S-3a-3b-E-M-N-7-3′ [6]. TGEV has both enteric and respiratory tropism, in contrast to other porcine enteric CoVs, making this virus a good model to analyze the molecular determinants of coronavirus tissue tropism.

Spike (S) protein is the mayor structural protein in CoV envelope and is involved in receptor binding and membrane fusion [7,8,9,10]. In addition, S protein is the main inducer of neutralizing antibodies and, indeed, many CoV vaccines are based in the expression of S protein or S protein domains [11,12,13,14]. We and others have demonstrated that, by modifying S protein sequence, CoV tropism is altered [15,16,17,18]. Moreover, species tropism could also be changed by introducing S protein sequences in the genomes of distantly related CoVs [19,20,21,22]. Previous work from our group, using targeted recombination between a respiratory helper virus (TGEV-PTV) and a minigenome derived from an enteric TGEV, showed that recombinant viruses containing mutations in the S gene region between nt 217 and 665 lack enteric tropism in vivo [16]. Moreover, the analysis of recombinant viruses between enteric TGEV-PUR46-MAD and respiratory TGEV-PTV, determined that nucleotides at positions 214 and 655 of S gene were determinants of the enteric tropism [15].

**Figure 1:**
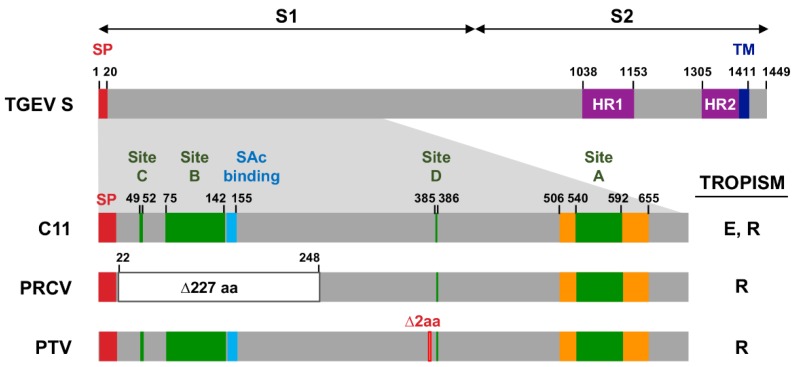
Transmissible gastroenteritis virus (TGEV) spike (S) protein domains. The bar in the upper part represents the S protein structure from a reference strain of an enteric TGEV (GenBank accession number AJ271965). The numbers above each bar indicate amino acid positions. S1 and S2 domains are indicated. SP, signal peptide (red); HR, heptad-repeat motifs (purple); TM, transmembrane domain (dark blue). The bars below represent a detail of N-terminus for enteric (C11) and respiratory [porcine respiratory coronavirus (PRCV), Purdue type virus (PTV)] TGEV-derived viruses, as previously defined in [16]. Several motifs are indicated: Antigenic sites C, B, D, and A (green) as previously defined in [9,28]; porcine aminopeptidase N (pAPN) binding domain (orange); Sialic acid (SAc) binding domain (light blue); large deletion (white rectangle); and in frame two amino acid deletion (red). E, enteric; R, respiratory.

Porcine respiratory coronavirus (PRCV) is a TGEV mutant that naturally appeared in the field, lacking enteric tropism [23,24]. PRCV contains a 227 amino acid deletion in the N-terminus of S protein (Figure 1), strongly suggesting that this region of TGEV S protein is important for the enteric tropism. In addition, we have previously shown that S protein binding to the host cell receptor, the porcine aminopeptidase N (pAPN), was not enough to determine the enteric tropism, as both TGEV and PRCV bind pAPN [10]. By comparing TGEV and PRCV binding to cell surface sialoglycoproteins, it was proposed that TGEV binding to this type of molecules may facilitate enteric tract infection [25]. Nevertheless, the sialic acid binding domain identified in TGEV S protein is conserved in several TGEV-derived respiratory viruses with a full-length S gene, such as TGEV-PTV (Figure 1), suggesting the existence of additional determinants of enteric tropism in the S protein. PRCV genome also contains deletions in the C-terminus of S gene, in genes 3a, 3b, and the ORF1a region encoding nsp3. Then, the contribution of other S protein domains or other viral proteins to enteric tropism was for a long time an open question [26]. In fact, the relevance of S protein N-terminus in TGEV enteric tropism has even recently been questioned [27].

Our group developed the first CoV infectious cDNA for TGEV, cloned in a bacterial artificial chromosome (BAC) [29]. Using the same TGEV genetic background, the tropism of the recombinant virus rescued from the infectious cDNA was manipulated by substituting the S gene from an enteric virus (rTGEV-SC11) by that from a respiratory isolate (rTGEV-SPTV) [30], with no additional changes in other locations of the viral genome. The newly engineered virus had only a respiratory tropism and lost the ability to infect the enteric tract [30]. It is worth noting that both rTGEV-SC11 and rTGEV-SPTV encode full-length S proteins (Figure 1), confirming that the determinants for TGEV enteric tropism rely on S protein and not in other viral proteins.

In this manuscript, taking advantage of the reverse genetics system allowing the generation of enteric (rTGEV-SC11) or respiratory (rTGEV-SPTV) recombinant viruses, the determinants of TGEV enteric tropism were mapped by extensively evaluating the tropism of all the generated viruses in the natural host, newborn piglets. The respiratory TGEV virus already contained the domains binding to pAPN (aa 506–655) and sialic acid (aa 145–155), which were necessary but not sufficient for enteric tract infection. Our data clearly demonstrated that the nature of the nucleotide at position 655 of S gene (leading to a S219A mutation in S protein) was required to confer enteric tropism to the respiratory TGEV. Moreover, a 6 nt insertion at position 1124 (leading to Y374-T375insND in S protein) led to a virus highly stable in cell cultures and increased by 1000-fold the virus titers in the enteric tract.

## 2. Results and Discussion

### 2.1. Engineering of TGEV Infectious cDNAs Expressing Chimeric S Protein

**Figure 2:**
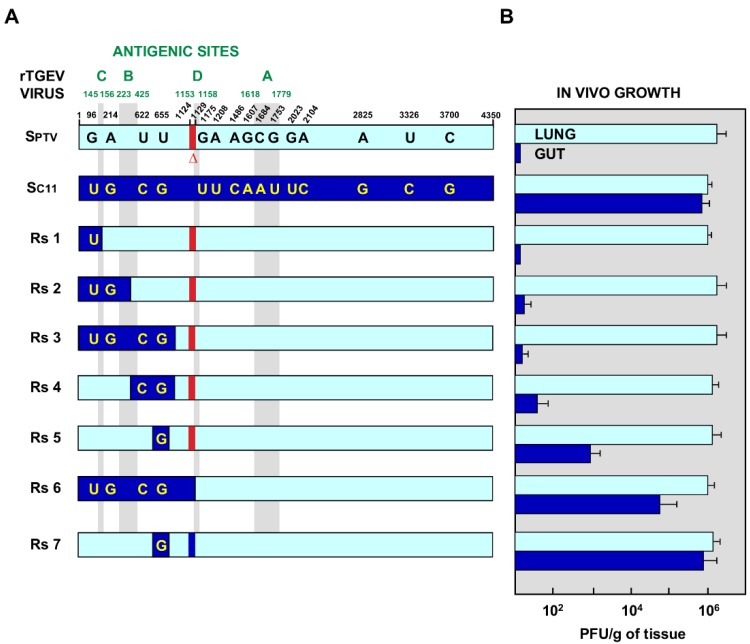
Engineered rTGEV-Rs mutants. (**A**) Chimeric recombinant TGEV viruses (Rs) were designed, based on the indicated nucleotide differences between S genes from respiratory PTV (light blue) and enteric C11 (dark blue) TGEV viruses. All mutant viruses encode an S protein derived from PTV, including different fragments from C11 S protein as indicated in the diagram. The location of antigenic sites C, B, D, and A, from the amino-terminus [9,28], is indicated in green, and the corresponding region in each Rs mutant protein is represented by the light gray bands. The red rectangle indicates an in frame two amino acid deletion. A dark blue rectangle in Rs7 indicates that the deletion was filled up with the corresponding sequence from the C11 S protein. Numbers in the upper part of the figure indicate the nucleotide positions. (**B**) Summary of rTGEV-Rs mutants tropism. Peak titers in lung (light blue) and gut (dark blue) for each recombinant virus. Data were obtained from three different animals. Error bars indicate the standard deviation for each value.

When the S genes (4350 nt) from an enteric (SC11) and a respiratory (SPTV) TGEV virus were compared, 17 nucleotide substitutions were identified and 15 of them leading to amino acid changes (Figure 2A). In addition, the SPTV contained an in-frame 6 nt deletion (Figure 2A). Based on previous data from our group [15,16], the attention was focused in the nucleotide substitutions located at the N-terminus of S protein. To identify the determinants for TGEV enteric tropism, a series of chimeric S proteins was designed, containing different SC11 domains into a SPTV background (Figure 2A). These mutants were introduced in the TGEV infectious cDNA, leading to the rescue of a set of recombinant viruses (rTGEV-Rs). All the rTGEV-Rs viruses were efficiently recovered, with titers in porcine swine testis (ST) cells ranging from 2 × 10^8^ to 5 × 10^9^ pfu/mL.

### 2.2. Growth of rTGEV-Rs Viruses In Vivo

**Figure 3:**
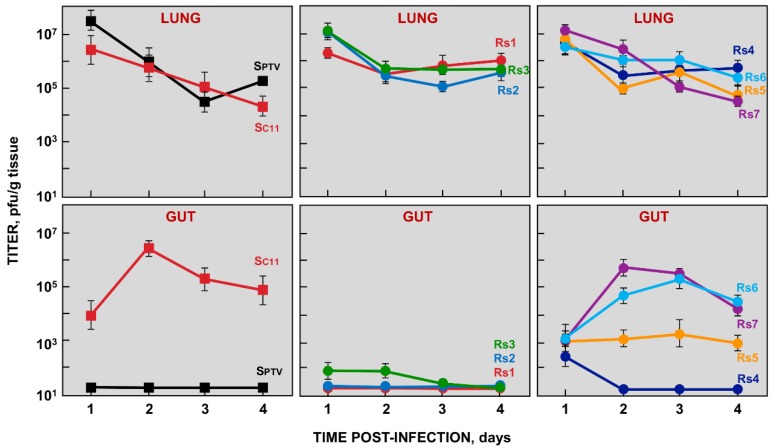
Growth of rTGEV-Rs mutants in vivo. Three-day-old piglets were inoculated with 10^8^ pfu/animal by nasal, oral and intragastric routes. Samples from lung and gut were recovered at the indicated days post-infection (dpi) and virus titers were determined in these samples. The values represent viral titers from three different animals. Error bars indicate the standard deviation for each value.

Three-day-old piglets were inoculated with 10^8^ pfu/animal of each rTGEV-Rs mutant, parental enteric rTGEV-SC11, and respiratory rTGEV-SPTV viruses as previously described [16,31]. Virus titers in the lung and intestine were analyzed at different days post-infection (Figure 3). As expected, the parental rTGEV-SPTV virus only replicated in the lung of infected piglets, while rTGEV-SC11 exhibited both enteric and respiratory tropism (Figure 3, left panels). The recombinant viruses rTGEV-Rs1, rTGEV-Rs2, rTGEV-Rs3, and rTGEV-Rs4 grew efficiently in the respiratory tract, but failed to grow in the enteric tract of the infected piglets (Figure 3, middle panels). Interestingly, rTGEV-Rs5, rTGEV-Rs6, and rTGEV-Rs7 grew both in respiratory and enteric tract (Figure 3, right panels), indicating that these viruses contained the S protein determinants of both enteric and respiratory tropism. It is worth noting that, in addition to the pAPN and sialic acid binding domains (present in all the rTGEV viruses engineered), the mutation U655 > G, leading to a S219A amino acid change, present in rTGEV-Rs5 virus was required for infection of the enteric tract (Figure 2B and Figure 3). Nevertheless, the addition of the 6 nt insertion at position 1124, leading to an Asn-Asp insertion between Tyr374 and Thr375 (Y374_T375insND), significantly increased virus growth in the gut, as rTGEV-Rs7 virus titers were 1000-fold higher than those of rTGEV-Rs5 virus (Figure 2B and Figure 3).

### 2.3. Stability of rTGEV-Rs Viruses

**Figure 4:**
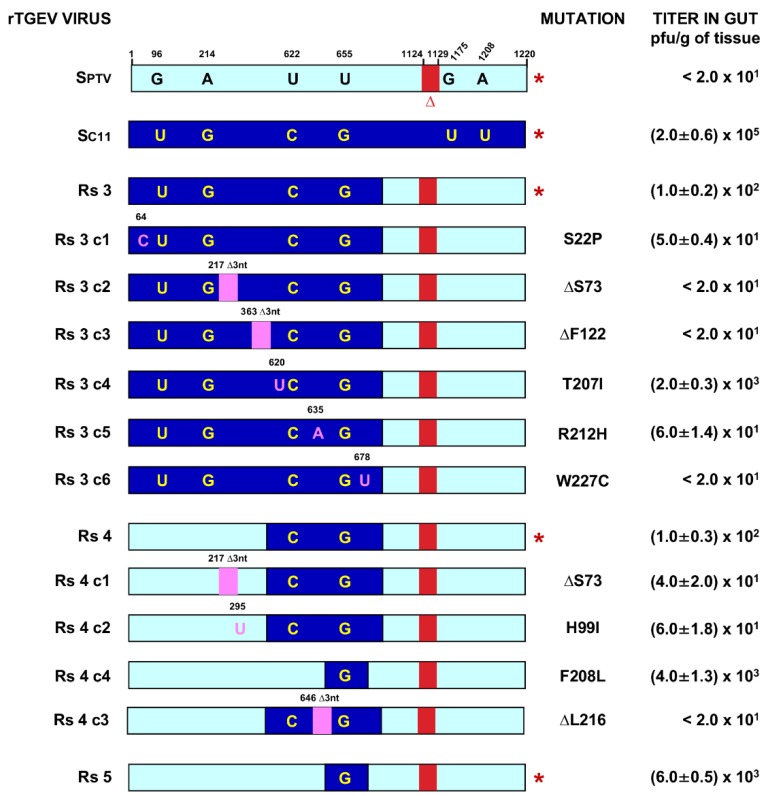
Summary of enteric tropism of viral clones isolated from tissues. The bars in the left panel indicate a detailed region (first 1220 nt) of the viruses analyzed, as in Figure 2. The red asterisk on the right indicates the engineered SPTV, SC11, Rs3, Rs4, and Rs5 protein sequences. Numbers in the upper part indicate the nucleotide positions. Letters in magenta indicate point mutations and deletions are indicate by the magenta box. The rest of the bars represent the sequences of isolated viral clones including spontaneous mutations. The columns at the right side of the bars indicate the amino acid changes, and the peak titers in gut for each recombinant virus. Data were medium values obtained from three different animals.

The data from rTGEV-Rs5 indicated that the presence of U655 > G, leading to a S219A amino acid change, was required for the infection of the enteric tract together with the presence of pAPN and sialic acid binding sites. In agreement with this observation, rTGEV-Rs6 and rTGEV-Rs7 also exhibited enteric tropism (Figure 2B). Nevertheless, U655>G was also present in rTGEV-Rs3 and rTGEV-Rs4 viruses, with an exclusive respiratory tropism (Figure 2B). Virus genetic instability may explain the differential behavior of rTGEV-Rs3 and rTGEV-Rs4 viruses compared with rTGEV-Rs5. To address this issue, independent clones were isolated from the viruses recovered from tissues and S gene was sequenced. It was observed that 100% of the isolated rTGEV-Rs3 virus clones included mutations and deletions in the SC11 inserted region (Figure 4). Each viral clone was then independently assayed in piglets, confirming that most of the recovered mutations (five out of six) led to viruses with exclusive respiratory tropism. Only one of the sequence variants (Rs3c4), representing one out of six viral clones, led to a virus showing partial enteric tropism with a titer in the enteric tract of 2 × 10^3^ pfu/mL, 100-fold lower than that of the enteric C11 virus (Figure 4). Similarly, 100% of rTGEV-Rs4 viral clones included spontaneous mutations (Figure 4). Most of these changes, representing three out of four viral clones, led to viruses with only respiratory tropism. Interestingly, one of the observed mutated clones, Rs4c4, led to a virus with the same sequence as that of the engineered rTGEV-Rs5 virus and grew in the enteric tract (Figure 4). These data confirmed that 655U > G mutation was one determinant of the enteric tropism, but that another factor was required for optimum replication in the enteric tract.

Further analysis of rTGEV-Rs6, with enteric tropism, showed that eight out of ten viral clones recovered from the enteric tract also contained mutations (data not shown), indicating that this virus was not stable. Interestingly, rTGEV-Rs7, which is the mutant virus reaching the highest titers in the enteric tract, was genetically stable in vivo, as all the viral clones contained the engineered sequence (data not shown).

To analyze virus stability in cell cultures and its effect on viral tropism, rTGEV-SC11 and rTGEV-Rs7 were passed eight additional times in cell cultures. Passage two after cDNA transfection (p2) and passage eight (p8) viruses were used to infect piglets and virus titers in intestine were determined. As previously observed, rTGEV-SC11 p8 virus had decreased virus titer in the gut compared with p2 (Table 1). In contrast, no significant difference in virus titer in enteric tract was observed between p2 and p8 rTGEV-Rs7 virus (Table 1). These data were further confirmed when rTGEV-Rs7 was used as an expression vector and virus was passed 16 times in cell cultures maintaining S protein sequence [32]. Therefore, Rs7 S protein sequence would be very useful to generate vaccine candidates that can be propagated in cell cultures without losing the enteric tropism and, therefore, maintaining all the immunogenic potential [1]. These vaccine candidates would be both for TGEV, including attenuating mutations [30], or for other viruses if TGEV is used as a viral vector [32].

### 2.4. Minimum Sequence Requirements for Enteric Tropism

In agreement with previous observations [16,30], our data indicated that the presence of sialic acid and pAPN binding domains was necessary but not sufficient for TGEV enteric tract infection. Additional requirements for enteric tropism were identified in this work. We have engineered a mutated rTGEV-Rs7 enteric virus that differs from a respiratory recombinant virus (rTGEV-SPTV) in just two locations in the S gene: U655>G and a 6 nt insertion at position 1124, leading to S219A and a Y374_T375insND insertion in S protein, respectively. It is worth pointing out that the 6 nt insertion was also present in the respiratory PRCV, although this virus lacks the sialic acid binding domain (Figure 1). Interestingly, comparing viruses containing both sialic acid and pAPN binding domains showed that, as occurs in respiratory rTGEV-SPTV virus, many TGEV enteric isolates contain a deletion at position 1124, such as strains WH-1 (GenBank HQ462571), USA/Z/1986 (GenBank KX900393), HX [33] or TH-98 (GenBank KU729220), strongly suggesting that this sequence alone is not a determinant of enteric tropism. By comparing our results on rTGEV-Rs5 and rTGEV-Rs7 viruses, it can be concluded that the presence of Y374_T375insND significantly increased the growth of TGEV in the enteric tract (around 1000-fold). In addition, it could also be strongly suggested that this sequence insertion may help virus stability in cell culture.

In contrast, U655>G mutation was a determinant of the enteric tract infection, as it was required for gut infection of a virus already containing sialic acid and pAPN binding domains. This mutation led to a S219A change in S protein, resulting in the elimination of a predicted glycosaminoglycan (GAG) attachment site. Then we postulated that, during adaptation to cell cultures, TGEV viruses acquire the U655>G mutation to increase GAG binding. GAG association with TGEV has been described [34,35]. And the use of GAGs as attachment factors in cell cultures has been described for other enteric CoV, the porcine epidemic diarrhea virus (PEDV) [36]. Very often, the interaction between viruses and GAGs traps virus progeny, inhibiting virus release and spread [37]. We hypothesized that the cell-culture adapted virus, containing the additional GAG binding site, may have decreased ability to traffic through the enteric tract to reach its target tissues and, as a consequence, has reduced virulence. Similar findings were described for other RNA viruses, such as foot-and-mouth disease virus (FMDV) [38], alphaviruses [39,40,41], and flaviviruses [42,43,44,45], reinforcing our postulate.

**Figure 5:**
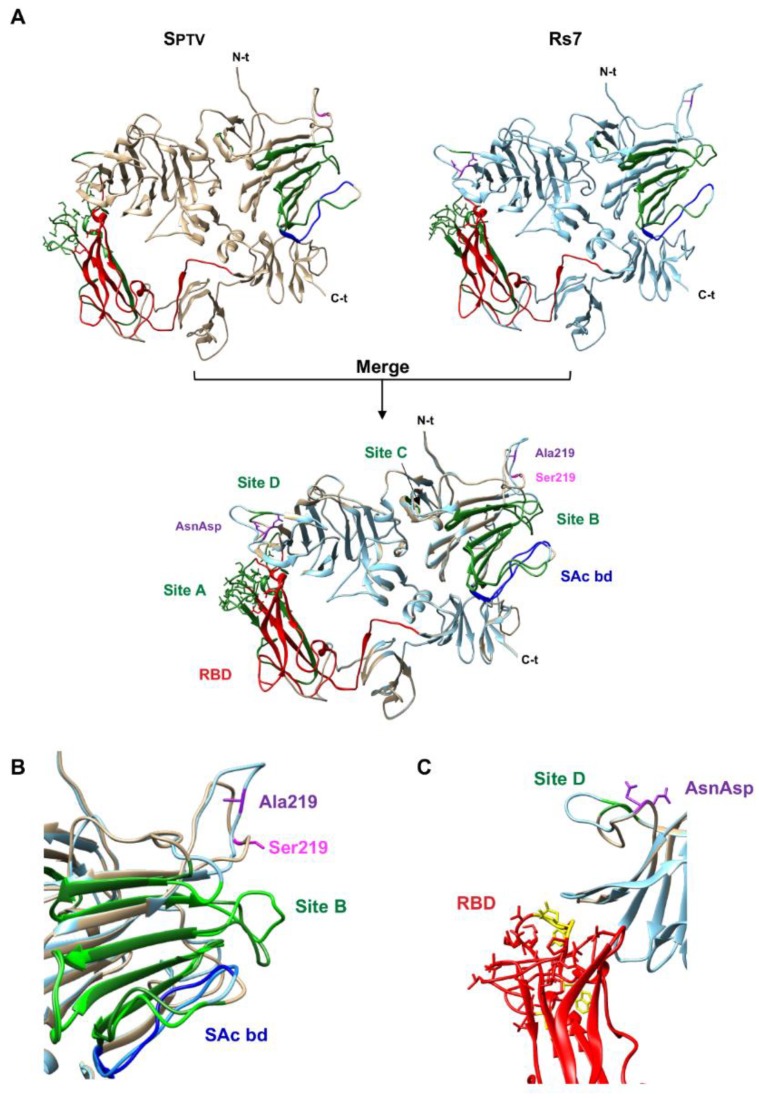
Comparison of SPTV and Rs7 structure models. (**A**) The S1 domains (aa 1 to 818) from SPTV (left panel, light brown) and Rs7 (right panel, light blue) were modelled and compared (merged structures in the bottom panel). Relevant domains are indicated, such as pAPN (receptor) binding domain (RBD, red), sialic acid binding domain (SAc bd, blue), and the antigenic sites A, B, C, and D (green). Site A partially overlaps with RBD. Ser219, present only in SPTV sequence, is indicated in magenta. Ala219 and the Y374_T375insND (AsnAsp), present only in the engineered Rs7 sequence, are indicated in purple. (**B**) Detail of the region around Ser219. Labels and colors as in panel A. (**C**) Detail of the region around the ND insertion in Rs7. The RBD residues involved in S-pAPN contacts are indicated, including those engaged in hydrogen bonding (in yellow) [10]. Labels and colors as in panel A.

There are 12 additional predicted GAG binding sites in the S1 domain of SC11 or Rs7, which are also present in the same domain of SPTV. This fact, together with the observation that non-engineered mutations introduced in vivo into the S1 domain of the spike protein of rTGEV-Rs3 and rTGEV-Rs4 viruses abrogated enteric tropism, suggests that structural constrains in the S protein of the N-terminal region may have a role in the determination of the enteric tropism. Unfortunately, the structural information on TGEV S protein is limited to the APN binding region (aa 506 to 655) (Figure 5A) [10], not allowing the location of S219A mutation in the experimentally determined S protein structure. As an alternative, S1 domains (aa 1 to 818) were modeled using RaptorX web server [46]. The prediction showed limited differences between the SPTV and Rs7 structures (Figure 5A). One of the biggest differences was in the vicinity of Ser219, changing the predicted orientation of the loop including aa 219 (Figure 5B). Moreover, this structural perturbation may also affect some loops in antigenic site B and the sialic acid binding domain (Figure 5B). Interestingly, the Y374_T375insND also may produce a structural change affecting antigenic site D and S protein loops which are close to the pAPN binding residues (Figure 5C). Therefore, it could be postulated that S219A and Y374_T375insND mutations, present in rTGEV-Rs7, may structurally favor interactions with host cell receptors.

Our results confirmed that the enteric tropism determinants are located in the N-terminus of TGEV S protein. Previously published data questioned the relevance of this domain in the enteric tropism [27]. It is worth noting that, in contrast to the data reported by other groups, we compared the tropism of engineered viruses containing a full-length S protein. Moreover, we concluded that the respiratory and the enteric viruses only differ in two positions: S219A and Y374-T375insND.

## 3. Materials and Methods

### 3.1. Ethics Statement

Experiments involving animals were performed in strict accordance with EU (2010/63/UE) and Spanish (RD 53/2013 and 32/2007) guidelines. All the protocols were approved by the in site Ethical Review Committee.

### 3.2. Cells

Baby hamster kidney cells stably transformed with the gene coding for porcine aminopeptidase N (BHK-pAPN) [47] were grown in in Dulbecco’s modified Eagle’s medium (DMEM) supplemented with 5% fetal calf serum (FCS) (Hyclone, Cultek, Madrid, Spain) and G418 (1.5 mg/mL) as selection agent. Recombinant TGEV viruses obtained in this work were grown in ST cells [48]. ST cells were grown in DMEM supplemented with 10% FCS.

### 3.3. Plasmid Constructs

Plasmids pGEMT-SPTV-1881, containing nucleotides 20,296 to 22,246 of TGEV-PTV genome, pGEMT-SC11-1881 and pGEMT-SMAD-1881, containing nucleotides 20,296 to 22,240 of TGEV-C11 and TGEV-PUR46-MAD genomes, respectively, were used for intermediate cloning steps [16]. Plasmid pGEMT-SC11-1881 was digested with NcoI and BsmI to get Rs1 fragment, with NcoI and NsiI for Rs2 fragment, with NcoI and MscI for Rs3 fragment and with NsiI and MscI for Rs4 fragment. Plasmid pGEMT-SMAD-1881 was digested with NsiI and MscI to get Rs5 fragment. All these fragments were cloned into the same restriction sites in pGEMT-SPTV-1881. The obtained plasmids were subsequently digested with NcoI and XhoI and the fragments were cloned in pGEMT-SPTV plasmid, containing nucleotides 20,287 to 24,811 of TGEV-SPTV genome and engineered PacI and MluI restriction sites [30], generating plasmids pGEMT-Rs1, pGEMT-Rs2, pGEMT-Rs3, pGEMT-Rs4, and pGEMT-Rs5. To obtain plasmid pGEMT-Rs6, pGEMT-SC11-1881 was digested with NcoI and XhoI and this fragment was cloned intro the same sites in pGEMT-SPTV plasmid. Plasmid pGEMT-Rs7 was obtained by overlapping PCR. Briefly, a 1095 bp PCR product was amplified using pGEMT-Rs5 as a template and oligonucleotides S-77(Pac) VS (5′-CCTTAATTAAGAAGGGTAAGTTGCTC-3′) and S998RS (5′-CGTCTACAGTATTATTTAAAACAGCACC-3′). A second 672 bp PCR product was amplified using pGEMT-Rs6 as a template and oligonucleotides S998VS (5′-GGTGCTGTTTTAAATAATACTGTAGACG-3’) and S1640RS (5’-GATGTTACTTAATGTTGAGG-3’). Both PCR products were combined and amplified using S-77(Pac) VS and S1640RS oligonucleotides. The obtained 1739 PCR product was digested with PacI and XhoI and cloned into the same sites in pGEMT-SPTV plasmid. Finally, pGEMT-RS plasmids were digested with PacI and MluI and the fragments were cloned into the same sites in pBAC-TGEV-SPTV [30] to obtain the corresponding infectious cDNAs. All cloning steps were checked by sequencing of the PCR fragments and cloning junctions. For each mutant sequence, two independent cDNAs were constructed. To rescue parental control viruses, pBAC-TGEV-SPTV and pBAC-TGEV-SC11 [30] were used.

### 3.4. Transfection and Recovery of Infectious rTGEVs from cDNA Clones

BHK-pAPN-N cells grown to 90% confluence in 35 mm plates were transfected using 4 µg of the corresponding pBAC and 12 μL of Lipofectamine 2000 (Invitrogen, Carlsbad, CA, USA) according manufacturer’s specifications. At 6 h post-transfection (hpt) BHK-pAPN transfected cells were trypsinized and plated over confluent ST monolayers grown in 35-mm-diameter plates. After a two-day incubation period, the cell supernatants were harvested (passage 0) [49]. The rTGEVs were cloned by three plaque purification steps, grown, and titrated as previously described [50].

### 3.5. Analysis of Viral RNA by RT-PCR

Total intracellular RNA was extracted from ST cells infected with rTGEVs and it was purified with RNeasy Mini kit (Qiagen, Hilden, Germany) according to the manufacturer’s specifications. Total cDNA was synthesized using 100 ng of total RNA as a template, random hexamers, and the High-capacity cDNA transcription kit (Life Technologies, Foster City, CA, USA), following the manufacturers’ instructions. A specific 1258 bp region of viral RNAs was amplified from the obtained cDNA using oligonucleotides S-69 VS (5′-GAAGGGTAAGTTGCTC-3′) and S1170 RS (5′-ACCGTGGTCCATCAGTTACG-3′) to analyze full-length S gene by sequencing.

### 3.6. In Vivo Growth Analysis

The in vivo growth of recombinant TGEV viruses was determined as previously described [16]. Briefly, groups of twelve three-day-old piglets obtained from TGEV seronegative sows were oronasally and intragastrically inoculated with 10^8^ pfu/animal of each recombinant TGEV virus in a BSL3 containment facility. A group of four animals was mock inoculated, as a negative control group. For viral titers determination, samples from lung, jejunum, and ileum were recovered from three animals at the indicated times post inoculation.

### 3.7. Linear Motifs and Protein Structure Predictions

The GAG attachment sites were predicted with ELM server [51]. These are short amino acid sequences with the pattern: (E/D)_0–3_ X S (G/A) X.

The structure of the S1 domain (aa 1 to 818) of SPTV, SC11, and Rs7 was modelled in silico using the RaptorX web server [46]. Modelling was performed in two domains: N-t domain (aa 1–241) and the C-t domain (aa 242 to 818), with an unnormalized global distance test (uGDT) and a *p*-value indicated in Table 2. The UCSF Chimera software was used for model visualization and comparison [52].

### 3.8. Statistic Analysis

Two-tailed, unpaired Student *t* tests were used to analyze the difference in mean values between groups. All results were expressed as means ± the standard deviations of the means. *p* values < 0.05 were considered significant.

## Figures and Tables

**Table 1:** Recombinant virus growth. Viral titers of non-passed and passed viruses in cell cultures and in vivo are shown.

	Cell Cultures ^1^		In Vivo			
	Titer (Pfu/ML)		Titer (Pfu/G of Tissue)			
			Lung		Gut	
Virus	P2 ^2^	P8 ^3^	P2	P8	P2	P8
rTGEV-SC11	(3 ± 0.7) × 10^7^	(4 ± 0.3) × 10^8^	(5 ± 0.7) × 10^6^	(2 ± 1.5) × 10^6^	(5 ± 1.3) × 10^6^	(5 ± 1.0) × 10^5^
rTGEV-Rs7	(5 ± 0.2) × 10^8^	(3 ± 0.5) × 10^8^	(4 ± 1.2) × 10^7^	(8 ± 2.9) × 10^6^	(8 ± 1.7) × 10^6^	(4 ± 5.1) × 10^6^

^1^ grown in porcine swine testis (ST) cells. ^2^ Passage two virus, obtained after transfection of infectious cDNA plus two additional passages in ST cells. ^3^ P2 virus was passed six additional times in ST cells, leading to P8.

**Table 2:** Quality parameters for the structural models.

	N-T Domain		C-T Domain	
S Protein	uGDT	*p*-Value	uGDT	*p*-Value
SPTV	105	1.0 × 10^−8^	400	7.6 × 10^−31^
SC11	106	1.4 × 10^−8^	410	2.0 × 10^−32^
Rs7	105	1.1 × 10^−8^	407	2.0 × 10^−31^
